# The Role of Nanovaccine in Cross-Presentation of Antigen-Presenting Cells for the Activation of CD8^+^ T Cell Responses

**DOI:** 10.3390/pharmaceutics11110612

**Published:** 2019-11-15

**Authors:** Cheol Gyun Kim, Yoon-Chul Kye, Cheol-Heui Yun

**Affiliations:** 1 Department of Agricultural Biotechnology and Research Institute for Agriculture and Life Sciences, Seoul National University, Seoul 08826, Korea; cjfrbs73@snu.ac.kr (C.G.K.); highspect@snu.ac.kr (Y.-C.K.); 2 Center for Food and Bioconvergence, Seoul National University, Seoul 08826, Korea; 3 Institute of Green Bio Science and Technology, Seoul National University, Pyeongchang, Gangwon-do 25354, Korea

**Keywords:** nanovaccine, cross-presentation, CD8^+^ T cell, cytotoxic T lymphocyte, cancer vaccine, dendritic cell

## Abstract

Explosive growth in nanotechnology has merged with vaccine development in the battle against diseases caused by bacterial or viral infections and malignant tumors. Due to physicochemical characteristics including size, viscosity, density and electrostatic properties, nanomaterials have been applied to various vaccination strategies. Nanovaccines, as they are called, have been the subject of many studies, including review papers from a material science point of view, although a mode of action based on a biological and immunological understanding has yet to emerge. In this review, we discuss nanovaccines in terms of CD8^+^ T cell responses, which are essential for antiviral and anticancer therapies. We focus mainly on the role and mechanism, with particular attention to the functional aspects, of nanovaccines in inducing cross-presentation, an unconventional type of antigen-presentation that activates CD8^+^ T cells upon administration of exogenous antigens, in dendritic cells followed by activation of antigen-specific CD8^+^ T cell responses. Two major intracellular mechanisms that nanovaccines harness for cross-presentation are described; one is endosomal swelling and rupture, and the other is membrane fusion. Both processes eventually allow exogenous vaccine antigens to be exported from phagosomes to the cytosol followed by loading on major histocompatibility complex class I, triggering clonal expansion of CD8^+^ T cells. Advancement of nanotechnology with an enhanced understanding of how nanovaccines work will contribute to the design of more effective and safer nanovaccines.

## 1. Introduction

Nanotechnology has been applied to various fields of medical research including vaccine development. It has contributed to the enhancement of efficacy and, more importantly, the safety of vaccine candidates. The advantages of nanosized particles (NPs), including upregulation of immune response, safety with biodegradability, tissue or cell target ability through size modulation, and conjugation with immune-regulatory factors, can be utilized at several stages of vaccine development. By harnessing these features, NPs have been applied to the development of effective vaccine delivery agents or immune-stimulating vaccine adjuvants [1,2].

Nanovaccines, consisting of NPs (as the antigen itself, adjuvant, or material for delivery/targeting), have attracted attention from the scientific community and health industry as they have been known to selectively induce humoral and/or cellular immune responses. Beyond the conventional antibody-inducing effects of vaccines, the role of the cellular immune response, represented by the activation of CD8^+^ T cells that directly kill infected or abnormal cells, has been highlighted recently in vaccines against viral infections and cancer. In addition, it has been well appreciated that some nanovaccines trigger cellular immunity, including CD8^+^ T cell responses [3]. On the other hand, it is never too much to emphasize the importance of understanding the working principles of vaccines with respect to enhancing their safety and efficacy and, at the same time, with minimizing their side effects [4]. Although nanovaccines provide a paradigm for the development of new vaccine platforms, their mechanism of action has not been fully elucidated. In this review, we will discuss the role of nanovaccines in inducing CD8^+^ T cell responses with a detailed mechanism.

Dendritic cells (DCs), one of the most effective professional antigen-processing cells (APCs), are required for bridging innate and adaptive immunities via antigen uptake, and for processing and presenting the epitope to naïve T cells. For appropriate CD8^+^ T cell responses from an exogenous antigen, DCs present epitopes from the antigen after loading its epitope on major histocompatibility complex (MHC) class I molecules to CD8^+^ T cells through a mechanism known as “cross-presentation” [5,6,7,8]. As most vaccines used in the field are exogenous to the cell, DCs have a critical role in vaccine inducing activation of cytotoxic T lymphocyte (CTL) responses against viral or cancerous diseases. For this reason, various strategies for nanovaccines have been designed to target DCs [2,9]. Studies have been carried out to unveil the mode of action of nanovaccines by focusing on intracellular components and their roles. Thanks to accumulated proof-of-concept studies of cross-presentation, novel nanovaccines have been suggested and designed to induce protective CTL responses.

Many review articles on nanovaccines dealt primarily with the types and physicochemical properties of nanomaterials. [10,11,12]. Although informative, the immunological context of these reports is not sufficient. In this review, by addressing the key concept of how nanovaccines activate CD8^+^ T cell responses, we discuss (1) how nanoparticles advance antigenicity and adjuvanticity to enhance effectiveness, (2) nanovaccines which target lymph nodes (LNs) and APCs, and (3) intracellular mechanism to harness cross-presentation of DCs.

## 2. Nanoparticles Incorporated with Antigens and Adjuvants for CD8^+^ T Cell Response

### 2.1. Nanoparticle Incorporated with Antigens

Physicochemical properties, especially the size of the nanomaterial, are important for the delivery and the efficacy of nanovaccines. The size of a nanovaccine and its surface area can improve antigen uptake and increase the probability of interactions with target cells compared with traditional vaccines. The size of the nanomaterial reportedly helps vaccine penetrate mucosal surfaces more easily [9,13] and may determine different immune responses. It has been reported that nanoparticles 20–200 nm in diameter are usually internalized via endocytosis into APCs that induce T cell responses, while 0.5–5 μm particles are mainly internalized by phagocytosis, which favors a humoral immune response [14,15]. Moreover, the surface charge of nanoparticles can also affect internalization of nanovaccines and play a critical role in antigen trafficking [16]. Cationic nanoparticles can be taken up rapidly by APCs and induce more efficient lysosomal escape that is important for cross-presentation [17]. In addition, reverting surface charge of nanoparticle from negative to neutral or positive, potentially induces protonation of the acidic groups on poly(lactide-*co*-glycolic) acid (PLGA) that can explain the lysosomal escape capacity of PLGA [18]. Managing the size and charge of nanovaccines is therefore important to inducing precise and efficient immune responses. In the same vein, various NP-formulating strategies have been introduced for nanovaccines to induce a CD8^+^ T cell response [19].

Incorporation of nanoparticles with antigens and/or adjuvants to improve efficacy of both prophylactic and therapeutic vaccines can be achieved by simple physical adsorption or more sophisticated methods, such as chemical conjugation or encapsulation [10,20]. In general, the absorption of an antigen with nanoparticles is based on charge or hydrophobic interactions [21,22]. A physical adsorption based on a simple mixture of a nanoparticle has a relatively weak interaction with the antigen that could easily result in disassociation from the nanoparticle, while chemical conjugation and encapsulations exhibit relatively strong interactions. For chemical conjugation, antigens are chemically cross-linked to the surface of nanoparticles [23]. Encapsulation refers to a form of encapsulating by mixing the interested antigenic molecules as a vaccine candidate with precursors of nanoparticles during synthesis of nanoparticles, resulting in enclosure of the antigen by nanoparticles [24]. For example, chitosan is a promising biomaterial for antigen encapsulation due to its ability to form nanoparticles in mild aqueous conditions that preserve the immunogenicity of loaded polypeptide antigens [25,26]. Encapsulin, originally isolated from thermophile Tehrmotoga maritima, is with inner and outer diameters of 20 nm and 24 nm, respectively, that self-assembles in crystal structures from 60 copies of an identical subunit and has an empty space in the center. Because of the cavity, encapsulin is thought to encapsulate functional proteins [27]. Such nanocarriers containing antigenic peptide showed excellent antigen-specific CTL response and tumor rejection via effective antigen delivery to DCs [28].

NP-facilitating technologies can contribute to enhancing the antigenicity of tumor vaccines, which requires CD8^+^ T cell responses. For therapeutic cancer vaccines, tumor cell lysate is considered a vaccine candidate because it offers the advantage of tumor-specific antigens from the patient’s own tumor without additional sequencing and/or synthesis of antigens. In addition, a tumor-cell lysate would allow the loading of a wide variety of antigenic molecules [29]. However, a therapeutic vaccine using tumor lysate is often insufficient and too weak to induce tumor-specific T cell responses followed by a low therapeutic effect. To overcome such limitations, incorporation of nanoparticles with tumor lysate has been attempted for tumor vaccines [30]. For instance, a simple method of generating nanovaccines with tumor cell lysate via polyethylene glycol (PEG)ylation, a process for both covalent and noncovalent incorporation with PEG, has been reported. Briefly, using autologous whole tumor cell lysate, tumor cell membranes were formed into monodispersed nanoparticles coated with a surface layer of PEG (PEG-NPs). The PEGylated tumor cell membrane vesicle induced an increase in antigen-specific cytotoxic CD8^+^ T cell responses after the vaccination as it has an efficient delivery ability into draining LNs and a stability through reducing interactions of PEGlyated antigens with phagocytic cells or serum proteins [31].

The other well-known NP forming strategy uses PLGA, a versatile nanoparticle approved by the US Food and Drug Administration (FDA). Due to its biodegradability and biocompatibility, PLGA has been widely applied as a nanovaccine [2]. It is synthesized through co-polymerization of glycolic acid and lactic acid. Many studies have demonstrated that PLGA incorporates with antigens of nanovaccines to induce CD8^+^ T cell response from different perspectives, such as size and formulation. PLGA nanoparticles were efficiently taken up by DCs for better MHC class I–mediated antigen presentation, resulting in improved antigen-specific CD8^+^ T cell responses compared with those of PLGA microparticles. [32]. On the other hand, antigen-encapsulated PLGA exhibited the ability to upregulate MHC I expression, resulting in efficient antigen presentation to CD8^+^ T cells, while antigen-absorbed PLGA preferred to enhance MHC II expression for helper T cell responses [33]. PLGA also played a role as a carrier of immunostimulators including Toll-like receptor (TLR) agonists such as cytosine-phosphate-guanine (CpG) [33,34], monophosphoryl lipid A (MPL-A) [35] and Poly I:C [32,36].

### 2.2. Nanoparticles Incorporated with TLR Ligands for Enhanced Adjuvanticity

Adjuvants for vaccine formulation can boost vaccine efficacy. Among them, aluminum salt has been used for approximately 80 years. Recently, two squalene-based oil-in-water adjuvants (AS03; and MF59) were included in FDA-licensed vaccines [37,38]. An in-depth understanding of the mechanisms involved in immunology has contributed to the design of protective and safe vaccines and adjuvants. In particular, the discovery of pathogen-associated molecular patterns (PAMPs) that specifically interact with pattern-recognition receptors (PRRs) on innate immune cells made it possible to design advanced adjuvants such as AS04, which consists of aluminum hydroxide and MPL-A, a TLR4 agonist [39,40,41,42].

Signaling through PRRs can enhance innate immune responses to induce expression of cytokines and co-stimulatory molecules, which are critical for the function of APCs to induce T and B cell responses more efficiently along with antigen presentation. TLR family ligands, which are the most extensively identified PAMPs, have been used to upregulate immune responses as adjuvants. Many researchers have also begun to employ TLR ligands in nanovaccines. At first, immune stimulators such as TLR agonists were introduced for simple mixing with nanovaccines without considering their interactions or administrated independently with antigens [10]. However, in recent studies, TLRs have been incorporated directly into nanoparticles.

Among several TLRs and their ligands, CpG oligonucleotide (ODN), one of the TLR9 agonists, has been widely used in nanovaccine formulation to trigger the cellular immune response of CD8^+^ T cells [43]. Given that TLR3, 7, and 9 reside mainly in endosomal vesicles, rather than on the cell surface membrane of APCs, their ligands fit well with nanovaccines, which are designed to enter through the cell membrane. For instance, polystyrene particles carrying CpG ODN induced different immune responses in DCs depending on their size. With changes in endosomal pH, nano-particulate CpG resulted in both interleukin (IL)-6 and interferon alpha (IFN-α) production, while micro-particulate CpG produced IL-6 only. The initial endosomal pH of DCs, where the larger CpG particulates reside, was higher than that of smaller CpGs. Furthermore, by employing endosomal acidification inhibitors, it became clear that a gradual increase of endosomal pH had a negative correlation with IFN-α production, while IL-6 production remained constant, suggesting that the size of the CpG particulate can affect the profile of cytokines via modulation of endosomal pH [44]. This can be advantageous for inducing a CTL response because type I IFN is known to induce cross-presentation of DCs [45] or directly affect the activation of CD8^+^ T cells for clonal expansion and memory formation [46].

On the other hand, there are at least four types of CpG ODN, namely D-, P-, K- and C-types. Each type has different backbones, sequences, and immunostimulatory properties [47], leading to both advantages and disadvantages. For example, structurally, D- and P-type CpG ODNs form a higher-order structure that appears to be necessary for localization at the early endosomes to interact with TLR9. However, D- and P-type CpG ODNs show polymorphisms, aggregation, and precipitation in production. So, it has been suggested that only K- and C-type CpG ODNs are preferred for human vaccines [48,49,50]. Because of the ambivalence of each CpG type depending on the purpose (i.e., type of vaccine, expected effector immune response and route of vaccination), efforts have been made to develop a versatile CpG ODN that induce proper immune response without aggregation. A nanoparticulate humanized CpG ODN (K3) wrapped by nonagonistic Dectin-1 ligand, schizophyllan (SPG), K3-SPG, has been reported for the strong induction of CTL responses with type I and type II IFN production. As an influenza vaccine adjuvant, K3-SPG showed protective effects in both murine and a nonhuman primate model [51]. In addition, intravenously injected K3-SPG efficiently induced tumor-specific cytotoxic CD8^+^ T cell responses further suggesting an anticancer immunotherapeutic effect [52].

With respect to the CTL response induced by nanovaccines, TLR7 and TLR3 agonists have been incorporated into nanoparticles. For example, nanovaccines encapsulating imiquimod, a TLR7 ligand, induced DC maturation by upregulating the co-stimulatory molecules, CD80, CD86, and MHC II [53,54]. Moreover, TLR7 and TLR3 exhibit distinct synergistic effects with other TLRs. Nanoparticulated-adjuvants involving double TLR stimulation had originally been emphasized for enhancing humoral immunity [55]. Synergistic effects of combining different TLR ligands have been reported for the induction of cellular immunity. For example, virus-like nanovaccines loaded with CpG ODN and combining imiquimod showed much greater vaccine efficacy together with effector and memory CD8^+^ T cell response in melanoma patients [56]. In addition, while no results were shown a T cell response directly, the combination of polyinosinic polycytidylic acid (poly I:C), TLR3 agonist, and CpG-loaded spiky gold nanoparticles (SGNPs) synergistically enhanced maturation of DCs with an increase of cytokine (IL-6 and TNF-α) release and co-stimulatory molecules (CD80, CD86, and CD40) [57]. In addition, a lipid-based nanoparticle containing a poly I:C–imiquimod complex showed excellent efficacy against a melanoma tumor model by triggering a protective immune response via LN-resident DCs provoking robust tumor-specific CD8^+^ T cell responses [58].

A TLR4 agonist, MPL-A, has also been applied to nanovaccines to induce cellular immune responses. MPL-A, a derivative of lipid A from *Salmonella minnesota* R595 detoxified via hydrolytic treatment, is the first PRR agonist adjuvant approved for use in human hepatitis B vaccines. [59,60]. Because TLR4 agonists are known to induce pro-inflammatory cytokines, including IL-1β and IL-18, through NK-κB pathway or type I IFN through interferon regulatory factor (IRF)-3 pathway in APCs, they have also been tried with nanovaccines. A co-delivery system using multiple target peptides (TRP180-188 and HGP10025-33) with a MPL-A adjuvant based on lipid-coated zinc phosphate hybrid nanoparticles (LZnP NPs) was investigated for antitumor immunity. In this system, the coordinative binding property of zinc phosphate contributes to the encapsulating ability and, at the same time, the lipid coating enhances incorporation capability of lipid-like adjuvant, MPL-A that showed effective anti-tumor-specific CD8^+^ T cell responses with IFN-γ expression [61].

Collectively, nanoparticles incorporated with antigens and TLR agonists enhance the efficacy of vaccine, particularly for cellular immune responses. Studies have suggested that the incorporation of nanoparticles with antigens and/or adjuvants could be achieved in various ways, from simple physical adsorption to chemical conjugation and encapsulation. The basic physicochemical properties of the nanoparticles have an important role to play in delivery and improved efficacy of vaccines with respect to the induction of CD8^+^ T cell responses. In-depth studies including those that specifically target immune cells or organs and harness the intracellular-level mechanisms, are underway.

## 3. Targeting Strategies with Nanovaccines for CD8^+^ T Cell Responses

### 3.1. Nanovaccines Targeting Lymph Nodes

Delivery of an antigen to target tissues and cells is an important element of the efficacy of vaccines and efforts to minimize side effects. Various nanovaccines containing an antigen and adjuvant, including PEGylated nanoparticles, PLGA-nanoparticles [62], and nanoparticles incorporated with TLR agonists [63,64] have shown improved abilities to target the LNs that activated LN-resident immune cells [65]. Cationized gelatin-based nanoparticles containing CpG selectively target LNs and activate the APC, resulting in protective antitumor effects associated with neither widespread systemic inflammation nor immunostimulation caused by free CpG [64]. In addition, poly I:C-encapsulating PLGA showed increased persistence of poly I:C in LNs, leading to prolonged DC activation and enhanced CD8^+^ T cell responses [62].

In general, effective delivery of vaccines to LNs and increased retention have been considered for appropriate CD8^+^ T cell responses. Intradermal delivery of functional pluronic-stabilized poly(propylene sulfide) nanoparticles showed an ability to target skin-draining LN and LN-resident DCs [66,67]. When coupled with an antigen and adjuvant, nanovaccines exhibited cross-presentation of DCs leading to enhancement of antigen-specific CD8^+^ T cell responses [68,69,70]. For example, an anticancer vaccine using a tumor-draining lymph node (tdLN)-targeting nanovaccine consisting of a tumor antigen showed robust cytotoxic CD8^+^ T cell responses, both locally and systemically, despite the immune-suppressed environment of tdLN [71]. However, how this effect was achieved by the nanovaccines is not fully understood.

It has been suggested that the size of a nanovaccine is strongly correlated with its efficiency to target the LNs. Through the interstitium, vehicles of vaccine enter lymphatic capillaries and then, drain into LNs. Because of the tight junction between endothelial cells, only molecules smaller than 10 nm wide can enter the blood capillaries. On the other hand, larger molecules can enter the lymphatic capillaries since lymphatic vessels have a discontinuous basement and inter-endothelial junction. In addition, for efficient delivery to an LN, its size is limited to 100 nm because molecules smaller than 100 nm can move through the interstitium. Although, molecules smaller than 10 nm easily enter lymphatic as well as blood capillaries, the flow rate is 100–500 times faster in blood capillaries. Therefore, particles between 10 and 100 nm appear to be optimal for delivery to LNs [65]. Given that the size of nanoparticles ranges from 1 to 100 nm in general [72], this interrelationship between the size of the nanovaccine and lymphatic system could explain the high efficiency of LN-targeting nanovaccines. However, molecules larger than 100 nm could still transfer to LNs after being phagocytosed by APCs.

On the other hand, nanovaccines can likely harness the recruitment of immune cells through modulating cytokines and chemokines, enhancing the chance for interactions between APCs and T cells. It has been suggested that ultra-small graphene oxide covalently modified with carnosine (Go-Car) could promote a robust and durable antigen-specific antibody response with proliferation and activation of both CD4^+^ and CD8^+^ T cells by modulating the expression of IL-6, chemokine ligands (i.e., CXCL1, CCL2), and CSF3 [73]. A tuberculosis vaccine containing nanoparticle-fusion protein complexes showed an increase of both humoral and cellular immunities, including CD4^+^ and CD8^+^ T cell proliferation, and tissue-resident memory T cell populations in the lung. Furthermore, the protective effect of nanoparticle-fusion protein complexes caused by IRF-3-associated APC activation was identified with upregulation of chemokines and receptor expression such as CCR7, CXCL10, and CCL5 [74]. Numerous studies on nanovaccines have dealt with expression of cytokines or chemokines and their receptors. The ability to modulate recruitment of immune cells by LN-targeting nanovaccines could explain its mode of action.

Not only the generation of antigen-specific effector T cells but also their migration to the target site is essential for therapeutic success. Chemokines orchestrate circulation, homing, and retention of immune cells through their chemotactic properties. It can also affect the function of T cells [75]. CCR7 plays an important role in both the homing of T cells and the trafficking patterns in lymphoid organs such as LNs and the spleen [76]. Naïve T cells express CCR7, while LN, Peyer’s patch-associated high endothelial venules and afferent lymphatic vessel constantly express CCL21, which is a CCR7 ligand. Through the chemotactic interaction of CCR7–CCL21, the recruitment of naïve T cells is regulated [77]. In addition, the interaction of CCR7–CCL21 is further engaged with the recruitment of DCs to afferent lymphatic vessels and LN. After the maturation of DCs, expression of CCR7 is upregulated while the expression of tissue-specific chemokine receptors such as CCR1, CCR5, and CCR6 is downregulated [78]. In addition, CCR5 and their ligands, CCL3 and CCL4, regulate naïve CD8^+^ T cell recruitment toward the site of antigen-specific interaction between DCs and CD4^+^ T cells. Immunization without CD4^+^ T cell activation showed a limited accumulation of antigen-specific CD8^+^ T cells when compared with immunization with CD4^+^ T cell activation. Both DCs and CD4^+^ T cells produce CCL3 and CCL4 after activation following the immunization, and CD8^+^ T cells isolated from draining LNs showed CCR5 expression. Indeed, either the knockout of CCR5 or neutralization of CCL3 and CCL4 abrogated the accumulation of CD8^+^ T cells to antigen-specific interaction site for DCs and CD4^+^ T cells [79].

### 3.2. Nanovaccines Targeting APCs

Despite insufficient understanding of the mode of action, it has been suggested that the effects of LN-targeting nanovaccines are ultimately initiated and mediated through APCs in the LN. For a vaccine to induce a protective immune response, the APCs should recognize the antigen and interact with adaptive immune cells. In other words, bridging the innate and adaptive immune system through proper antigen presentation by APCs is a crucial step toward maximizing vaccine efficacy. DCs, among APCs, specialize in presenting antigens and priming naïve T cells. Thus, there are a large number of studies of nanovaccines with an aim to induce protective immune responses by targeting DCs. For example, one study detailed a polymeric dissolving microneedle array loaded with a nano-encapsulated antigen was designed to specifically target Langerhans cells that showed efficient cross-priming and Th1 immune responses [80]. The cationic dendrimer-like α-d-glucan nanoparticle is reportedly available for adsorption of protein antigens, enhancing delivery of antigen to DCs and activating DCs, including cytokine secretion such as IL-1β and IL-12 [81]. Distribution and clearance of the α-d-glucan nanoparticle and its interaction with immune cells were examined. As a result, α-d-glucan nanoparticles were shown to remain at the injection site and cleared gradually. The α-d-glucan nanoparticle can deliver antigen to APCs, especially targeting immuno-potentiating migratory DCs [82].

DCs express cell surface-associated mannose receptors to efficiently internalize extracellular antigen via mannosylation, which leads to enhanced activation of CD4^+^ and CD8^+^ T cell response [83]. A vaccine strategy using mannose receptor-mediated internalization has become a well-established option for DC-target vaccination [84,85]. For CD8^+^ T cell response, nanosized dextran (DEX) particles have been applied to a DC-targeting nanocarrier platform due to the mannose receptor-mediated uptake ability of DCs. A DEX-based nanovaccine with lipopolysaccharide (LPS), a TLR4 agonist, showed robust antigen-specific CD4^+^ and CD8^+^ T cell responses. It showed a stronger induction of CD8^+^ T cell responses compared with a soluble form of the antigen and LPS [86]. In addition, beyond the general mannosyl ligands, gold nanoparticles that contained mannose-mimicking shikimoyl for targeting DCs, showed enhanced vaccine efficacy compared with mannosyl ligand to deliver vaccines to DCs via mannose receptor-mediated manner after the immunization. This nanovaccine significantly inhibited melanoma growth and enhanced overall survival rate in melanoma-challenged mice in a therapeutic vaccination strategy [87]. Dendritic cell-specific intercellular adhesion molecule-3-grabbing non-integrin (DC-SIGN), a well-characterized C-type lectin receptor, also has a specificity to high mannose and Lewis-type glycan. Targeting DC-SIGN on DCs by using its ligands has improved the uptake and processing of antigen together with the presentation of the epitope to antigen-specific T-cells [88]. Furthermore, Langerin (CD207) is exclusively expressed on Langerhans cells, which are a subset of DCs residing in the epidermis of the skin. Langerin-mediated targeting showed improvement of the endocytosis and cross-presentation, leading to CTL responses [89]. In a recent study, a novel specific glycomimetic Langerin ligand, which is conjugated with liposomes showed an ability to induce specific and efficient targeting of Langerhans cell in human skin [90].

As discussed above, several nanovaccines have been designed to target LNs and APCs. In fact, nanovaccine targeting of APCs has also been established with relatively rational modes of actions using distinct receptors, such as the mannose receptor, DC-SIGN and Langerin. Indeed, LN-target nanovaccines are well established and studied, but how their target abilities are achieved remains unclear. Although the size of the nanovaccine plays a critical role in delivery of vaccines to LN, it does not explain the mechanism of nanovaccines and their efficacy. A more comprehensive understanding based on immunology can provide a better explanation, leading to potent and safe nanovaccines. Further studies that reveal the exact mode of action for the tissue-targeting ability of nanovaccines are required.

## 4. Cross-Presentation and Cytosolic Exportation of Nanovaccines

**Figure 1:**
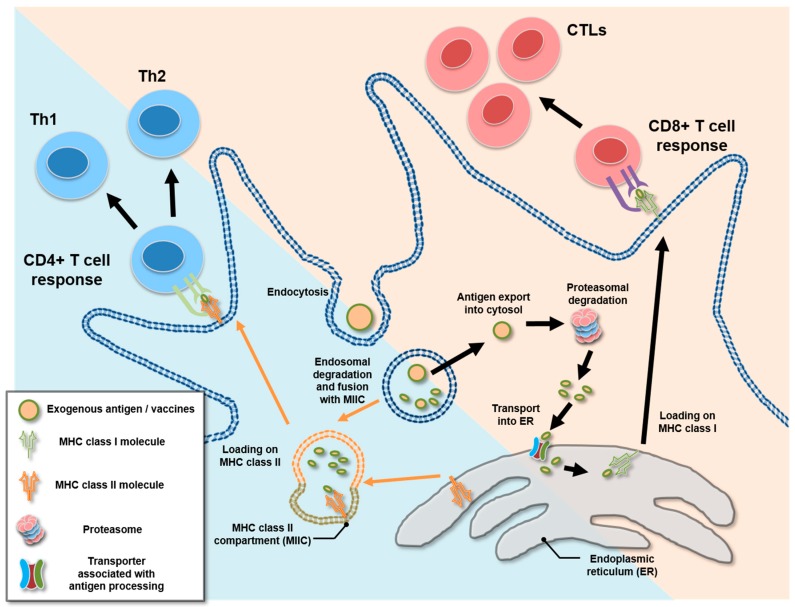
Schematic diagram for the presentation of an exogenous antigen in DCs. The exogenous antigen, in a general process, is presented by an MHC class II loading pathway (cyan color background). After endocytosis, the endosome-containing antigen goes through the process of maturation, including acidification. Then, MHC class II-containing intracellular vesicles called the MHC class II compartment fuses with the endosome. Through this process, the antigen degraded by proteolytic enzymes and loaded onto MHC class II leads to the presentation to CD4^+^ T cells. Alternatively, an exogenous antigen could be presented with MHC class I via cross-presentation (yellow background). After endocytosis, the antigen could be exported into cytosol via various pathways (see details in Figure 2). The exported antigens (epitopes) degraded by proteasome are transported to endoplasmic reticulum via transporter associated with antigen processing (TAP). Then, the epitope is loaded onto the pocket of MHC class I resulting in recognition by and activation of CD8^+^ T cells.

Beyond delivering antigens to specific tissues and cells, nanovaccines could modulate intracellular antigen delivery, securing the position of the epitopes during antigen processing and presentation by APCs. Nanovaccines may induce a change of property on DCs to present exogenous antigenic molecules to CD8^+^ T cells via a distinct pathway called “cross-presentation”. Generally, exogenous antigens are loaded onto MHC class II molecules resulting in CD4^+^ T cell responses while endogenous antigens loaded on MHC class I molecules result in CD8^+^ T cell responses. However, through cross-presentation, exogenous antigens are loaded onto the pocket of MHC class I molecules, resulting in the activation of CD8^+^ T cells. In other words, exogenous antigens can be presented not only via classical MHC class II pathways but also, under certain circumstances, MHC class I through cross-presentation (Figure 1). Considering that most vaccines are administrated as exogenous antigen, cross-presentation can play a major role in the initiation of CD8^+^ T cell responses especially against cancers and other infectious diseases caused by intracellular pathogens [5,8]. Major cross-presentation occurs via the cytosolic pathway, where processing of an antigenic molecule onto an MHC pocket occurs in the cytosol. In this pathway, exogenous antigens are exported from endosomal vesicles to the cytosol for degradation by the proteasome in the cytosolic pathway [5,7]. Originally, cytosolic delivery was focused in order to increase the efficiency of transfection of plasmids or nucleotide vaccines by delivering them to the cytosol of target cells [91,92,93]. In recent years, due to improved understanding of immunological mechanisms, including cross-presentation and antigen-trafficking in intracellular compartments, cytosol exportation of antigenic molecules has attracted attention in the area of vaccinology, where activation of CD8^+^ T cells is essential [8]. As a result, nanovaccines that can induce cytosol exportation for proper cellular immune responses have been designed. Among them, some polymer-based nanovaccines that induce endosomal swelling and rupture and liposome-based pH-sensitive nanovaccines showed notable achievements against viral or cancerous diseases in which the CD8^+^ T cell response is an integral part (Table 1).

### 4.1. Proton Sponge Effect and Photochemical Internalization of Polymer-Based Nanovaccines

#### 4.1.1. Proton Sponge Effect

**Figure 2:**
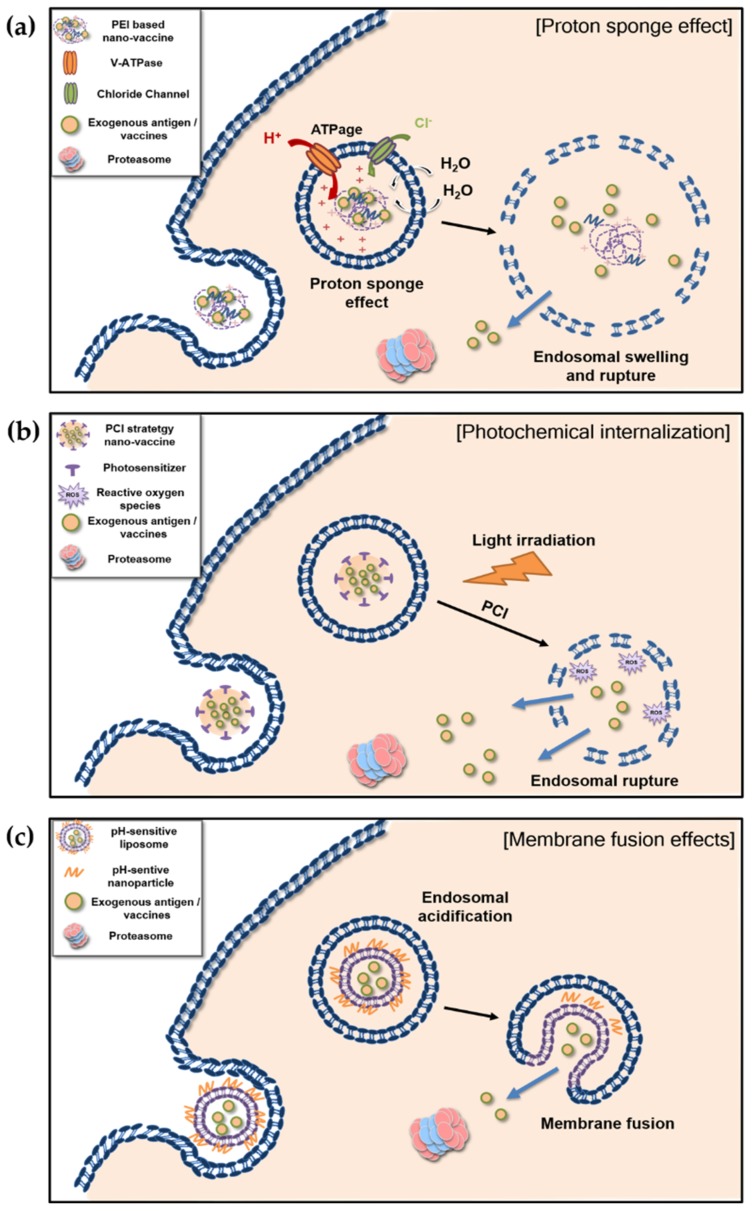
Schematic representation of the mechanism of a nanovaccine inducing cytosolic exportation, leading to cross-presentation: (**a**) proton sponge effect, (**b**) photochemical internalization, (**c**) membrane fusion effect of pH-sensitive liposome. (**a**) After cationic PEI-based nanovaccines are taken up, and an unsaturated amino group buffers the influxing proton, supplied by a proton pump, v-ATPase. The proton pump continuously transports the protons into endosomes, which results in retention of chloride ions and water. Eventually, osmotic swelling and rupture of endosomes expose the antigen and vaccine compartment to the cytosol; (**b**) Photochemical internalization based nanovaccines contain a photosensitizer responding to a specific wavelength of light. After light irradiation, the photosensitizer initiates of reactive oxygen species, leading to rupturing of endosomes and cytosolic exports; (**c**) After endocytosis of liposomes combine with pH-sensitive nanoparticles, early endosome become acidic along with endosomal maturation. The acidic condition destabilizes the pH-sensitive liposomes, leading to enhanced membrane fusion with export of antigens into cytosol.

Polyethylenemine (PEI) is a nonviral vector for the transfection of nucleotides (i.e., DNA, RNA, siRNA, and plasmids). PEI’s ability to buffer influent protons, thereby inducing osmotic swelling and rupture of endosomes, is called the proton sponge effect. [107,108,109,110,111]. Briefly, protons, supplied by a proton pump (v-ATPase), are sequestered via protonation of profound unsaturated amino groups. The sequestering of protons results in retention of chloride ions and water, eventually leading to osmotic swelling and rupture of endosomes (Figure 2a). Swelling and rupture of endosomes that contain antigens by the proton sponge effect exposes the antigens to cytosol. The proton sponge effect has therefore been suggested as a candidate for increasing cross-presentation. A PEI-based approach to vaccine delivery and making adjuvants is often presented as a promising strategy for the formulation of a vaccine that induces proper CD8^+^ T cell responses [94,95,96].

PEI could form nanoparticles with antigens via electrostatic interaction, enhancing the cross-presentation ability of DCs. [94], Moreover, cationic alginate PEI (AP) nanogels can be stabilized with 3,3′-dithiobis(sulfosuccinimidylpropionate), a disulfide-containing cross-linker (SS) to reduce toxicity of PEI, generating a bioreducible AP nanogel (AP-SS). Both AP-SS and AP nanogel without a disulfide bond showed facilitated antigen uptake with enhanced MHC II antigen presentation. However, only the AP-SS appeared to induce upregulation of MHC I antigen presentation and CD8^+^ T cell mediated tumor protection, along with enhanced antigen-specific antibody production. [95]. This indicates that bioreducibility is an important property of PEI-based nanovaccines with respect to its mode of action, including antigen degradation and cytosolic release in DCs. In addition, PEI-modified aluminum hydroxide nanoparticles showed an increase of internalization and the release of antigen into cytoplasm of DCs with enhanced expression of co-stimulatory molecules (CD80 and CD86) and cytokine (IL-12) secretion. Combined with tumor-derived autophagosomes (dribbles) as antigens, enhanced tumor-specific T cell responses have been seen in both in murine and human T cells [96]. Another PEI-based nanovaccine, polysorbitor transporter (PST), which is prepared from sorbitol diacrylate and low-molecular-weight PEI via nasal vaccination, showed a safe and efficient immune response against respiratory syncytial virus and pneumonia infection. PST in particular exhibited a long-term immune response of more than 200 days for antigen-specific antibody formation [97]. Such an immune response of PST, causing proton sponge effects, induced DCs to interact with follicular helper T cells [98]. Furthermore, via the proton sponge effect, PST can enhance cross-presentation and antigen-specific CD8^+^ T cell immune responses. In this regard, studies are underway to apply PST as a novel vaccine agent to anticancer therapy. In addition to PEI-based nano-materials, other polycations can induce the proton sponge effect [109]. Endosomal swelling and rupture by proton sponge effects could be used in other polycation-based vaccine strategies for intracellular delivery. For example, a dual-sensitive, micelle-tailored vaccine (ovalbumin-loaded pH/redox dual-sensitive micellar vaccine, OLM-D) designed by conjugation of the antigen with amphiphilic poly(l-histidine)-poly(ethylene glycol) showed a proton sponge effect similar to that of PEI. It appears that cascade delivery of an antigen by OLM-D occurs in three steps: (1) fast redox-triggered antigen release from lysosomes; (2) increase of micelle disassembly; (3) rapid lysosome escape via the proton sponge effect. Through cascade delivery, including proton sponge effects, OLM-D induced upregulated and prolonged LN accumulation with enhanced CD8^+^ T cell responses, including IFN-γ secretion [99].

#### 4.1.2. Photochemical Internalization

Photochemical internalization (PCI) is another efficient and specific strategy for cytosolic exportation via endosomal rupture induced by light [112,113]. Delivering a photosensitizer to the endocytic vesicle of a cell is a key factor for PCI. The photosensitizer responds to specific wavelengths of light, leading to the initiation of reactive oxygen species and the rupturing of endosomes and cytosolic exportations [114,115] (Figure 2b).

Tetraphenyl chlorine disulfonate (TPCS2a), a well-established photosensitizer, has been utilized for PCI-meditated cytosolic antigen export. Through the PCI ability, TPCS2a can shift antigen processing and presentation from MHC class II to class I using various strategies such as TPCS2a-OVA complex, TPCS2a-liposome, and TPCCS2a-loading PLGA. This upregulation of cross-presentation using TPCS2a-enhanced CD8^+^ T cell response and IFN-γ expression and showed antitumor effects. [100,101,102,116,117]. In addition, pheophorbide A (PheoA), a hydrophobic photosensitizer, has been reported as a PCI-inducing nanovaccine. The nanovaccine consisting of a PheoA-PEI complex and OVA (PheoA-PEI/OVA NPs) exhibited enhanced of antigen exportation to cytosol following by light irradiation. When applied to DC2.4 cells, its cross-presentation ability to antigen-specific B3Z T cells was upregulated after light irradiation. It also showed an inhibitory effect on an in vivo tumor model [103]. In addition, arginine- and phenylalanine-based polyester amides (Arg-Phe-PEA(AP) nanoparticles) formed an electrostatic complex with AlPcS2, another photosensitizer that has been suggested for PCI to enhance CD8^+^ T cell responses. Indeed, AlPcS2 induced an increase of antigen uptake and photochemical interruption of endocytic compartments that enabled light-facilitated endosomal export of antigen, leading to an enhanced CD8^+^ T cell response [118].

These polymer-based vaccines, having proton sponge effects or photochemical internalization, can induce cytosolic transport of antigens through endosomal swelling and rupturing that accelerates upregulation of cross-presentation and therefore CD8^+^ T cell responses. Practical use of nanovaccines that induce endosomal swelling and rupturing via proton sponge effects or PCI could be a feasible strategy for vaccines against cancer and infectious diseases caused by intracellular pathogens where the CTL response is crucial.

### 4.2. Membrane Fusion of Liposome-Based pH-Sensitive Nanovaccines

Liposome-based nanomaterials have been used as vaccine delivery agents since 1974 [119]. As liposomes are nontoxic, biodegradable, and available in various sizes and capable of encapsulation, they have been regarded as excellent candidates for delivery agents for both vaccine and adjuvant [2,120]. Moreover, liposome-based nanomaterials can induce cytosolic export of antigens via either membrane fusion or membrane disruption [121]. Although some studies have suggested that liposome-based nanomaterials can display both fusion and disruption effects, the majority dealt mainly with the membrane fusion. Indeed, membrane fusion could be considered as unique property of liposome-based nanovaccines because the endosomal disruption effect originated from in other proteins or nanoparticles involved in liposomes, while membrane fusion effects result from the property of the liposome itself.

Membrane fusion is initiated by the recognition or contact of membranes, which pull closer and destabilize, initiating a mixing of lipids from both membranes. Not only intracellular process during subcellular compartmentalization, cell growth, and secretion of hormones and neurotransmitters but also invasion of target cells by enveloped viruses requires rapid, targeted, and regulated membrane fusion. It could occur between the cells, between intracellular compartments, plasma membranes, and/or between lipid-associated structures such as viral particles and cellular membranes [122]. Enveloped viruses utilize various proteins to induce membrane fusion for invading target cells. For instance, influenza virus hemagglutinin, viral fusion protein fuses with the endosome to form a fusion active trimeric structure [123]. Other viral fusion proteins have been introduced to liposome modification, resulting in enhanced membrane fusion of nanovaccines. The viral fusion protein-containing the liposome showed an ability to merge with endosomal membranes and induce cytosolic export of antigen leading antigen-specific cellular immunity [121,124,125]. However, vaccines containing these viral proteins may have been able to induce off-target effects, such as unintentional immune responses originating from viral molecules [105,126]. Considering both safety and efficiency, various synthetic molecules have been applied in various ways in hopes of improving the membrane fusion effect to make it adjustable for liposome-based nanovaccines [104,127,128,129,130].

Liposomes combined with a pH-sensitive nanoparticle, that destabilized in acidic condition and induces membrane fusion have been considered for a potential strategy for vaccine delivery. They are known to protect entrapped antigens against extracellular environment such as proteases and other serum proteins until being taken up by APCs. [105,126,131]. Early endosomes or phagosomes containing internalized antigens go through a maturation process in which endosomal or phagosomal pH decreases and proteolytic activity increases [132,133]. Such conditions could induce membrane fusion after a decrease of endosomal pH, leading to effective and adjustable cytosolic export of antigens (Figure 2c).

Among the various pH-sensitive liposomes, surface modification of egg yolk phosphatidylcholine liposome with a pH-sensitive poly(glycidol) derivative can generate distinct pH-sensitive membrane fusion abilities in weakly acidic condition because of carboxylation of poly(glycidol) together with various kinds of acid anhydrides [104,128,134,135]. A poly(glycidol) derivative based pH-sensitive liposome, 3-methylgultarylated poly(glycidol) (MGlu-PG), containing carboxyl groups with hydrophobic spacer moiety could generate potent pH-sensitive liposomes as cytosolic delivery agents via their powerful fusion ability [104]. MGlu-PG has been studied as a delivery agent in vaccine strategies for cytosolic export in many ways, including modification of the backbone structure, cationic lipid inclusion, and incorporation of polysaccharides. Results of a study that constructed MGlu-PG with two different backbone structures such as a hyperbranched form (MGlu-HPG) and linear form (MGlu-LPG), suggested that the backbone structure of pH-sensitive polymers can affect its interaction ability with membranes and its pH sensitivity [134,135]. Another study that modified pH-sensitive liposomes with cationic lipid incorporation using 3, 5-didodecyloxybenzamidine showed improved pH sensitivity in weakly acidic conditions in DCs. The cationic, lipid-incorporated, pH-sensitive liposome could deliver entrapped antigens to an endosome/lysosome as well as cytosol, leading to upregulation of both MHC class I- and II-mediated antigen presentation [106]. A pH-sensitive polymer has also been applied to polysaccharides such as dextran. Generation of pH-sensitive dextran with 3-methylglutarylated residue (MGlu-Dex) produced a highly pH-sensitive liposome with cytosolic export ability. Subcutaneous administration of the MGlu-Dex modified pH-sensitive liposome efficiently induced both humoral and cellular immunity with antigen specificity [105]. These modifications of an MGlu-based, pH-sensitive liposome commonly showed an efficient antigen delivery to cytosol and antitumor effects when administrated for cancer immunotherapy.

For cross-presentation in DCs, modulation of the maturation process is essential because excessive proteolysis does not favor cross-presentation but facilitates presentation via MHC class II [133,136]. Mild and limited degradation favors cross-presentation via cytosolic export for extended periods [5,6,137,138]. From this perspective, using a pH-sensitive-liposome with fusion ability in weakly acidic conditions could induce cytosolic escape before excessive maturation of the endosome or phagosome in more acidic conditions. It could therefore prevent excessive degradation of an antigen and simply transport it to cytosol. Although no studies connect this phenomenon directly to the pH-sensitive-liposome, it could be considered as harnessing the physiological condition such as maturation of endosome and phagosome. Moreover, the range of the acidity at, during, and after membrane fusion should be considered an important parameter. DCs are known to have a relatively decreased proteolytic capacity among APCs and a lower speed of endosomal maturation compared with that of other phagocytes, such as macrophages and neutrophils, which are much more specialized in phagocytosis and clearance of extracellular macromolecules rather than cross-presentation. Such tendencies are more apparent in distinct DC subtypes that have a specialized intracellular machinery for cross-presentation, such as CD8^+^ DCs or CD103^+^ DCs [5,6,7,8]. However, a majority of the reported studies dealt with pH-sensitive liposomes or other nanovaccines harnessing the cross-presentation and did not report a correlation with such a distinct subtype of DCs or its intracellular properties. While several studies of nanovaccines aimed at the CTL response suggest cross-presentation of DCs as its mechanism and a key role for the enhancement of efficacy, an in-depth understanding of DC subsets for cross-presentation, its intracellular pathways and key regulatory factors is still needed for the better design of nanovaccines.

## 5. Conclusions

Emerging nanovaccine studies have prompted the scientific community to understand the immunological actions needed to improve vaccine efficiency and safety, and to prevent the side effects that dictate the therapeutic success of a nanovaccine. Application of nanotechnology to vaccinology has succeeded in enhancing the effectiveness of vaccines, and the introduction of nanovaccines has produced a new paradigm for vaccine development. However, studies of their function and the precise mechanisms are relatively insufficient. Undoubtedly, maximizing safety and minimizing all possible side effects stem can form a comprehensive understanding of their action mechanism.

Recently, the fields of both immunology and nanotechnology have grown rapidly in recent years, and efforts to reveal a mode of action for nanovaccines in a context of protection at the molecular and cellular level have significantly increased. Studies for inducing CD8^+^ T cell responses against viral or cancerous diseases are especially notable. With the sincere hope that these efforts will lead to better and safer vaccine development, in this review, we discussed the application principle of nanovaccines that aim at the CTL responses by harnessing the properties of nanoparticles to enhance antigenicity and adjuvanticity or specifically target the lymph nodes and APCs. Furthermore, we suggest cross-presentation as the key mechanism for the activation of CTL when exogenous antigens are an option for the vaccine candidate. Given that most vaccines use exogenous antigens, the cross-presentation of DCs is the most important and valuable target for CTL response. Remarkable scientific understanding with advancement of technologies in the area of immunology together with nanomaterials is the major contributor to vaccine development. In the near future, the concept of cross-presentation in APCs in conjunction with CD8^+^ T cell activation will likely consolidate effective vaccines against devastating incurable cancers and intracellular pathogens.

## Figures and Tables

**Table 1:** Nanovaccines inducing cytosol exportation for cellular immune response.

	Applied Nanomaterial	Targeted Antigen	Host Species	Type of Evaluation Model	Delivery Method	Ref.
**Proton sponge effect**	PEI	Ovalbumin (OVA)	Mouse	In vitro		[94]
	Cationic alginate PEI nanogel with 3,3′-dithiobis (AP-SS)	OVA	Mouse	In vitro/In vivo	i.p.	[95]
	PEI-modified aluminum hydroxide	Tumor derived autophagosomes (DRibbles)	Mouse	In vitro/In vivo	s.c. (DC-based vaccine)	[96]
	PEI-based polysorbitor transpoter (PST)	OVA	Mouse	In vitro/In vivo	i.n.	[97]
	PEI-based polysorbitor transpoter (PST)	Pneumococcal surface protein A (PspA)	Mouse	In vitro/In vivo	i.n.	[98]
	Amphiphilic poly(l-histidine)−poly(ethylene glycol)	OVA	Mouse	In vitro/In vivo	i.p./s.c.	[99]
**Photochemical internalization**	TPCS2a	OVA	Mouse	In vitro/In vivo	i.d.	[100]
	TPCS2a-PLGA	OVA	Mouse	In vitro/In vivo	i.v.	[101]
	TPCS2-Liposome	OVA	Mouse	In vitro/In vivo	i.d.	[102]
	PheoA-PEI	OVA	Mouse	In vitro/In vivo	i.v. (DC-based vaccine)	[103]
	AlPcS2	OVA	Mouse	In vitro/In vivo	i.d.	[103]
**Membrane fusion**	MGlu-PG	OVA	Mouse	In vitro/In vivo	i.n./s.c.	[104]
	MGlu-Dex	OVA	Mouse	In vitro/In vivo	s.c.	[105]
	Cationic lipid-incorporated MGlu	OVA	Mouse	In vitro/In vivo	s.c.	[106]

Note: i.p., intraperitoneal; s.c., subcutaneous; i.n., intranasal; i.d., intradermal; i.v., intravenous.
